# Theoretical Study on Decomposition Mechanism of Insulating Epoxy Resin Cured by Anhydride

**DOI:** 10.3390/polym9080341

**Published:** 2017-08-04

**Authors:** Xiaoxing Zhang, Yunjian Wu, Xiaoyu Chen, Hao Wen, Song Xiao

**Affiliations:** 1 School of Electrical Engineering, Wuhan University, Wuhan 430072, China; wuyunjian@whu.edu.cn (Y.W.); wenhao198711@163.com (H.W.); xiaosongxs@gmail.com (S.X.); 2 State Grid Shaoxing Power Supply Company, Shaoxing 312000, China; xiaoyuwhu@163.com

**Keywords:** anhydride, epoxy resin, decomposition, simulation

## Abstract

High temperatures caused by partial discharge results in the decomposition of insulating epoxy resins in electrical equipment. In this paper, the ReaxFF force field is used to investigate the decomposition process of epoxy resins cured by anhydride and the formation mechanisms of small-molecule gases. Results show that the initiation reaction is the cleavage of an ester bond linked with an epoxy resin. Produced by the decomposition of ester groups, CO_2_ is the first and most abundant product. Meanwhile, CH_2_O can be generated through three main ways, although the process still depends on the decomposition of epoxy functional groups. H_2_O is produced by free radical collision and dehydration. The production of small-molecule gases has the following sequence: CO_2_, CH_2_O, CO, and H_2_O. The produced gases have the following order according to amount: CO_2_, CH_2_O, H_2_O, and CO.

## 1. Introduction

Anhydride-cured epoxy resins are widely used as solid insulators in large electrical equipment because of their excellent resistance to leakage traces [1]. Despite these features, these resins are prone to defects such as grooves and gas gaps during their manufacture, transportation, and operation because of electric, heat, and mechanical stresses. These defects render the surface of epoxy resins vulnerable to partial discharge, which can lead to an average temperature rise of 170 °C and a maximum of 1000 °C around a volume of 5 × 10^−11^ cm^3^ on the epoxy resin surface near the partial discharge area. Consequently, the epoxy resins are degraded. In particular, the surfaces of the epoxy resins become scabrous and gradually form grooves, thereby increasing the possibility of breakdown caused by the development of an electric tree in the epoxy resins [2,3,4,5]. Moreover, the products combine with water or oxygen in the equipment, thereby producing acidic substances, which not only dramatically increases surface conductivity but also speeds up the deterioration of epoxy resins [6,7]. Thus, studying the decomposition process of anhydride-cured epoxy resins is of great significance to the exploration of electric tree formation and the aging process of insulating resins. 

There have been many researchers investigating the decomposition mechanism of epoxy resin [8,9] and the prevention of decomposition by enhancing its thermal conductivity [10,11]. Vlastara et al. [12] studied the decomposition process of epoxy resin cured by hexahydrophthalic anhydride heated with laser light. They found that the process produces large amounts of CO_2_ and H_2_O and certain amounts of acetylene, acetone, and other small volatile substances. Gao et al. [13] confirmed that gas generation occurs during the thermal aging process of epoxy-cast busbar. Hudon et al. [14,15,16] studied the liquid and solid matters formed on the surface of epoxy resins under partial discharge conditions, and subsequently determined that the solid matter is calcium oxalate. 

The ReaxFF reactive force developed by Duin and Dasgupta et al. [17] defines the band angle and torsional force as a function of bond order and estimates the breakage between atoms on the basis of the relationship between bond length and bond order. Bond energy and bond order effectively simulate molecular reactions. The computational speed and accuracy of traditional quantum chemistry are sufficient for predicting the geometries, energies, and vibration energies of small molecules; however, these parameters are inadequate for exploring macromolecules and solid dynamic performance [18]. The ReaxFF force field can offset this disadvantage and has thus been widely used in small-molecule systems [19,20], polymer systems [21,22], high-energy material systems [23,24], metal oxide systems [25,26], and transition metal catalyst systems [27,28]. The thermal process of epoxy resin in printed circuit boards was investigated by ReaxFF force field. The first gas product was found to be CH_2_O, and other main small-molecule products include H_2_O, CO, and H_2_ [29]. Zhang et al. simulated the decomposition process of epoxy resin heated by microwave by using ReaxFF, and they investigated the factors influencing the H_2_O and H_2_ production rates [30]. 

In this paper, the ReaxFF force field was used to analyze the thermal decomposition process of epoxy resin cured by acid anhydride at different angles. Several representative small-molecule gas products were then studied to produce a theoretical basis for the aging process and theory of solid insulation in electrical equipment.

## 2. Simulation Details

**Figure 1:**
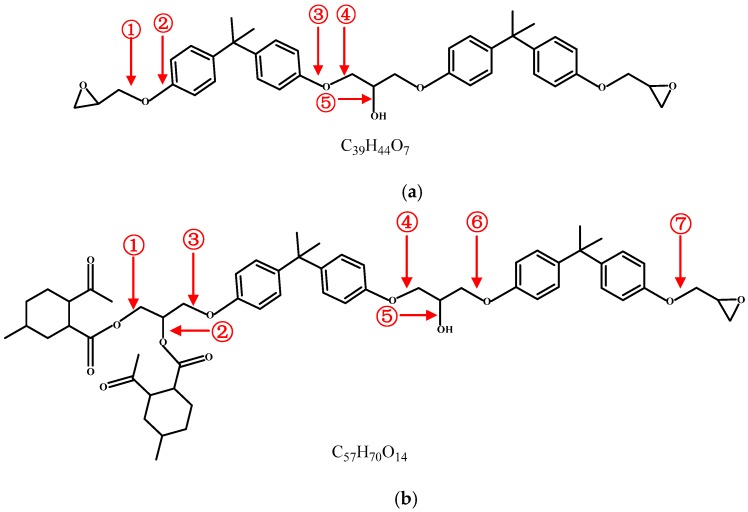
Structure of epoxy resin. (**a**) Structure of pure epoxy resin; (**b**) Structure of epoxy resin cured by anhydride.

In this paper, three models of non-cross-linked epoxy resin were built. Module 1 contains one pure epoxy resin (PEP) molecule, which is formed by the dehydration condensation of two bisphenol A diglycidyl ether molecules, as shown in Figure 1a (①②③④⑤ represent the carbon-oxygen bonds). Module 2 consists of one molecule of epoxy resin cured by methyl hexahydrophthalic anhydride (EPMHA), as shown in Figure 1b. Module 3 contains 15 EPMHA molecules, as shown in Figure 1b.

The first step is to build three-dimensional periodic models, the initial densities of which were all set to 0.5 g/cm^3^. Annealing via an iterative procedure was then performed, beginning with a molecular dynamics calculation by NVT ensemble at 600 K and then by NPT ensemble at 600 K and a constant pressure of 1.01 MPa (1 atm) for 100 ps; in the succeeding iterations, the temperature was decremented by 50 °C and the process was repeated until the specified temperature for the dynamics was 300 K (around room temperature). After which, geometry optimization of 5000 iterations was performed to get a more stable structure. The final densities of the three models were 1.13, 1.13, and 1.17 g/cm^3^ (Table 1). Subsequently, the NVT ensemble was selected, and the simulation temperature was set to 1300 K, which was the highest temperature during the real partial discharge. The time step and production time were then set to 0.1 fs and 1000 ps, respectively, to simulate the cook-off process.

**Figure 2:**
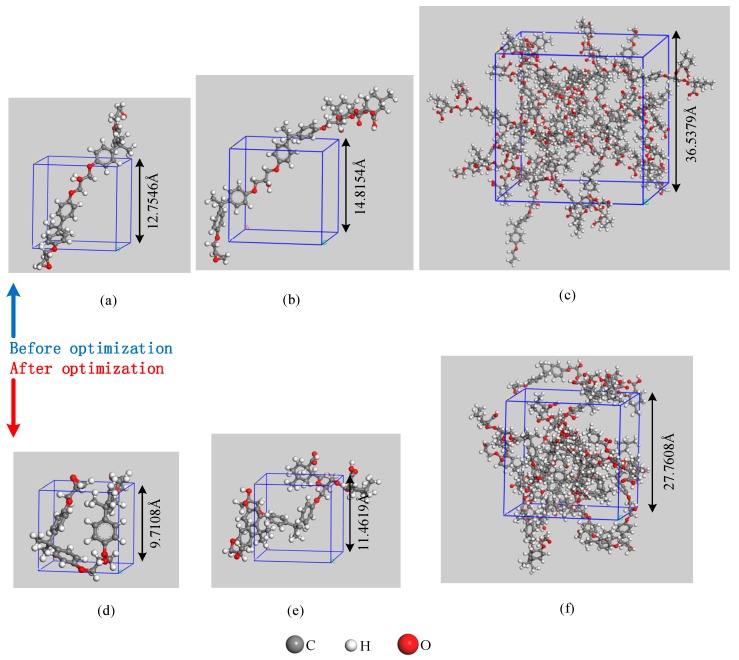
Structures of the three models. Structures before optimization of (**a**) Model 1, (**b**) Model 2, and (**c**) Model 3; Structures after optimization of (**d**) Model 1, (**e**) Model 2, and (**f**) Model 3.

The structures before and after the optimization of the three models are presented in Figure 2. Module 1 was used to study the process of PEP bond breakage under high temperatures, and the results were compared with the previous research results to verify the accuracy of the test program. Module 2 was used to simulate the bond-breaking process of EPMHA. The decomposition products of EPMHA during the cook-off process were obtained in Model 3. The decomposition mechanism of EPMHA and the formation of the corresponding products were analyzed by combining Models 2 and 3.

## 3. Results and Discussion

### 3.1. Simulation Results of PEP

**Figure 3:**
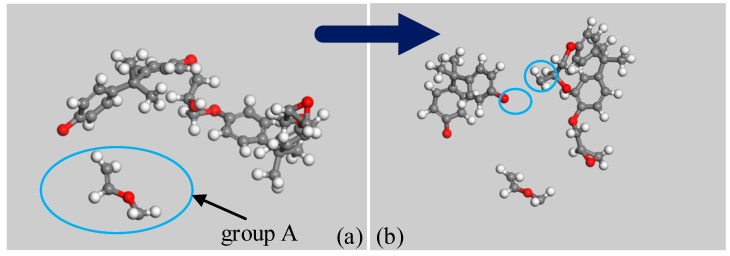
PEP decomposition process. (**a**) The first decomposition process; (**b**) The second decomposition process.

The carbon-oxygen bonds in epoxy resins tend to break, as shown in Figure 1a ①②③④⑤. According to Huckel’s rule, parts with aromatic structures exhibit better thermal stability than those without, and the activation energy required for the fracture is higher, as shown in at Table 2. Thus, the ② and ③ carbon-oxygen bonds are difficult to fracture. Among ①④⑤, the ① carbon-oxygen bond has the lowest activation energy. Thus, the breaking of this bond likely initiates PEP decomposition as shown in Figure 3a, and then results in the production of unstable intermediate product, Group A, which turns into vinyl radical and formaldehyde. The next breakage is observed in the ④ carbon-oxygen bond. All these findings agree with the conclusion of the initiation reaction and first gas product of the decomposition of PEP [25].

### 3.2. Simulation Results of EPMHA

**Figure 4:**
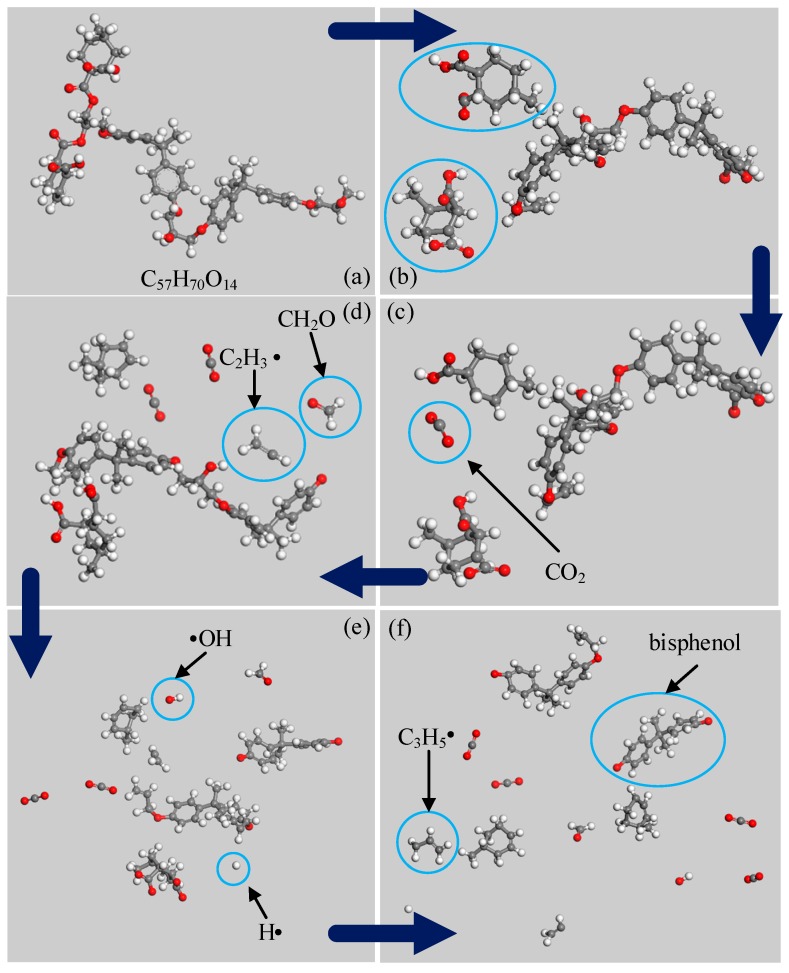
Decomposition process of EPMHA. (**a**) The first decomposition; (**b**) The second decomposition process; (**c**) The third decomposition process; (**d**) the forth decomposition process; (**e**) The fifth decomposition process; (**f**) The sixth decomposition process.

Figure 4 shows the simulation results of Model 2. Figure 4b shows that the initiation reaction of EPMHA decomposition is the cleavage of ① and ② ester bonds. The reason is that the ester group linked with α-C and hydrogen atom linked with β-C are in the same plane under high temperatures. These components form a six-atom center that facilitates elimination. Thus, the breakage activation energy of ① and ② are the lowest, as shown in Table 2. As shown in Figure 4c, the first gas product is CO_2_, and this result is consistent with the conclusion in this paper [8]. CO_2_ is mainly derived from acyloxy groups in acid anhydride. The ⑦ carbon-oxygen bond (Figure 1b) then breaks and forms vinyl radical and formaldehyde (Figure 4d). This process is the main mode of formaldehyde production. As shown in Figure 4e, the reaction produces free hydroxyl and free hydrogen because of the breakage of the ⑤ carbon-oxygen bond Figure 1b. The combination of free hydroxyl and hydrogen is one of the main approaches to producing H_2_O. Figure 4f shows that propylene and bisphenol free radical appear in the system at the final simulation. Owing to the limitation of the simulation temperature and time, the epoxy resin molecules cannot decompose completely, and no hydrocarbon formation is observed

**Figure 5:**
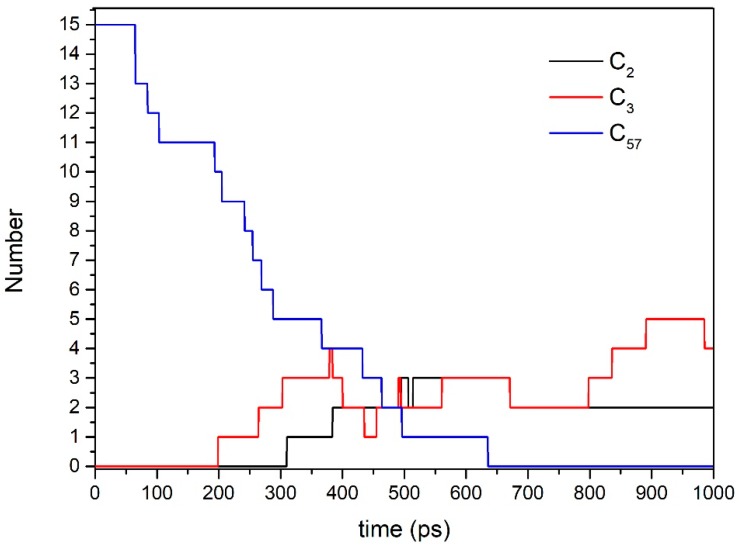
Time evolution of C_57_, C_2_, and C_3_.

**Figure 6:**
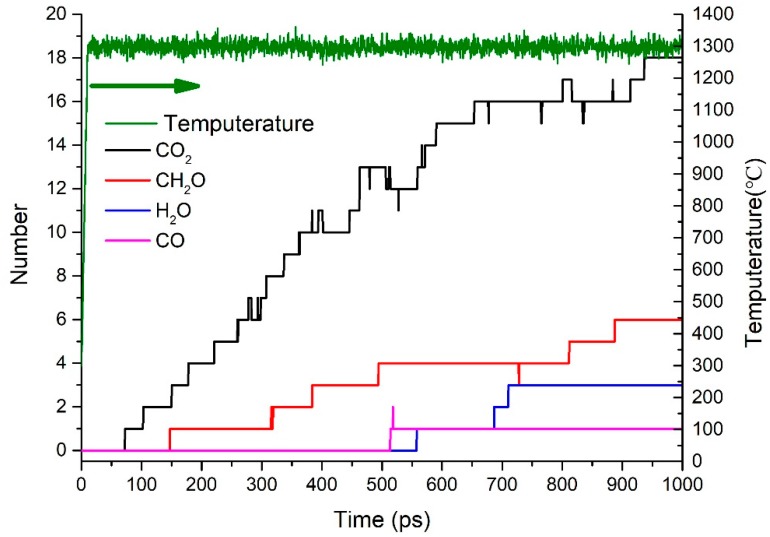
Time evolution of CO_2_, CH_2_O, H_2_O, CO, and temperature.

Figure 5 and Figure 6 are the results of Model 3. The changes in compounds C_57_, C_2_, and C_3_ over the simulation time is shown in Figure 5. C_57_ is a reactant. The main chain of the epoxy resin molecule starts to break from about 65 ps, and all the main chains of epoxy resin molecules are broken until 640 ps. The C_2_ products mainly include ethylene radicals and acetaldehyde radicals, and the C_3_ products contain propylene radicals, acetone radicals, acrolein radicals, propylene alcohol radicals, and so on.

Figure 6 shows the changes in CO_2_, CH_2_O, H_2_O, and CO contents over the simulation time. The first gas product is CO_2_ at about 70 ps, followed by CH_2_O and H_2_O. Thus, this result agrees with those of Model 2. The most abundant gas product is CO_2_ because of the numerous acyloxy groups. The limitations in this paper include the simulation temperature and time result in a small amount of CH_2_O and H_2_O, and the absence of H_2_ and CH_4_. The amount of hydroxyl groups in the model is comparable to the number of epoxy functional groups. However, the amount of CH_2_O is larger than that of H_2_O because the activation energy to form CH_2_O is lower than that of H_2_O.

### 3.3. Formation Mechanism of Gas Products

**Figure 7:**
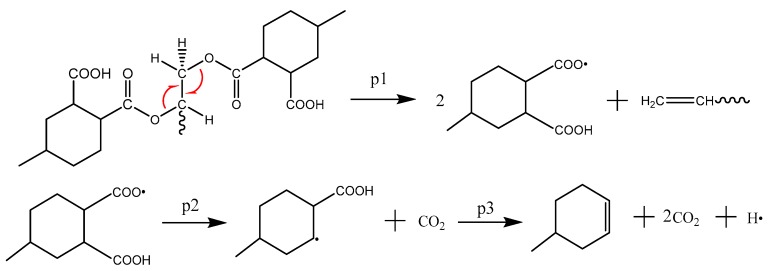
The main mechanism of CO_2_ formation.

**Figure 8:**
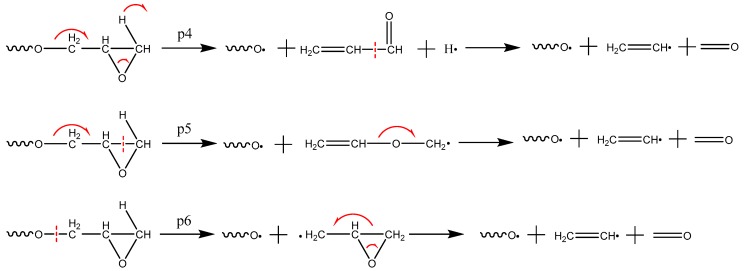
The main mechanism of CH_2_O formation.

**Figure 9:**
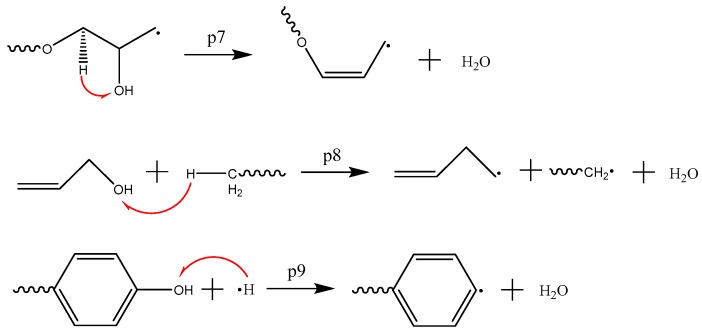
The main mechanism of H_2_O formation.

The main mechanism of CO_2_ formation is p1~p3, as shown in Figure 7. The first step is the cleavage of the ester bond, followed by the removal of the acyloxy bond in the acid anhydride. Cycloolefin then forms. As shown in Figure 8 (p4~p6), CH_2_O can be generated in three ways because the right-hand side of the ⑦ carbon-oxygen bond in Figure 1b can be converted through three different conversion methods. The water generation method includes three kinds. The p7 and p8 (Figure 9) indicate that the intra-molecular elimination reaction produces water and intermolecular dehydration reaction, and p9 represents the water-producing collision of free radicals.

## 4. Conclusions

In this paper, the ReaxFF force field was used to simulate a number of epoxy resin models, and the decomposition process of epoxy resin cured by anhydride and the formation path of main gas products were analyzed. The conclusions are as follows:

1. The initiation reaction of the decomposition of anhydride-cured epoxy resin is the cleavage of an ester bond;
2. The first and the most abundant product is CO_2_. The other products were generated in the following sequence: CH_2_O, CO, and H_2_O. With respect to content, the following order was observed: CH_2_O, H_2_O, and CO.
3. CO_2_ is produced from the carbonyl oxygen bond in anhydride; CH_2_O, from the decomposition of epoxy functional group; H_2_O, by free radical collision and dehydration.

## Figures and Tables

**Table 1:** Model construction. EPMHA: epoxy resin cured by methyl hexahydrophthalic anhydride; PEP: pure epoxy resin.

Model	Material	Molecular formula	Number	Initial density g/cm^3^	Final density g/cm^3^
Model 1	PEP	C_39_H_44_O_7_	1	0.5	1.13
Model 2	EPMHA	C_57_H_70_O_14_	1	0.5	1.13
Model 3	EPMHA	15*C_57_H_70_O_14_	15	0.5	1.17

**Table 2:** Activation energy of some carbon-oxygen bonds.

Molecule	Breakage of bond	Activation energy (kJ/mol)
PEP	①	−232.64
	②	−332.53
	③	−320.62
	④	−261.75
	⑤	−276.54
EPMHA	①	−192.27
	②	−186.58
	③	−256.81
	④	−258.14
	⑤	−273.92
	⑥	−252.38
	⑦	−230.78
